# High-Temperature Chemical Stability of Cr(III) Oxide Refractories in the Presence of Calcium Aluminate Cement

**DOI:** 10.3390/ma14216590

**Published:** 2021-11-02

**Authors:** Tengteng Xu, Yibiao Xu, Ning Liao, Yawei Li, Mithun Nath

**Affiliations:** 1 The State Key Laboratory of Refractories and Metallurgy, Wuhan University of Science and Technology, Wuhan 430081, China; xutengteng1992@163.com (T.X.); liaoning@wust.edu.cn (N.L.); 2 National-Provincial Joint Engineering Research Center of High Temperature Materials and Lining Technology, Wuhan University of Science and Technology, Wuhan 430081, China

**Keywords:** Al_2_O_3_-CaO-Cr_2_O_3_-O_2_ system, (Al_1−x_Cr_x_)_2_O_3_, Ca(Al_12−x_Cr_x_)O_19_, Cr(VI) compounds, leaching test

## Abstract

Al_2_O_3_-CaO-Cr_2_O_3_ castables are used in various furnaces due to excellent corrosion resistance and sufficient early strength, but toxic Cr(VI) generation during service remains a concern. Here, we investigated the relative reactivity of analogous Cr(III) phases such as Cr_2_O_3_, (Al_1−x_Cr_x_)_2_O_3_ and in situ Cr(III) solid solution with the calcium aluminate cement under an oxidizing atmosphere at various temperatures. The aim is to comprehend the relative Cr(VI) generation in the low-cement castables (Al_2_O_3_-CaO-Cr_2_O_3_-O_2_ system) and achieve an environment-friendly application. The solid-state reactions and Cr(VI) formation were investigated using powder XRD, SEM, and leaching tests. Compared to Cr_2_O_3_, the stability of (Al_1−x_Cr_x_)_2_O_3_ against CAC was much higher, which improved gradually with the concentration of Al_2_O_3_ in (Al_1−x_Cr_x_)_2_O_3_. The substitution of Cr_2_O_3_ with (Al_1−x_Cr_x_)_2_O_3_ in the Al_2_O_3_-CaO-Cr_2_O_3_ castables could completely inhibit the formation of Cr(VI) compound CaCrO_4_ at 500–1100 °C and could drastically suppress Ca_4_Al_6_CrO_16_ generation at 900 to 1300 °C. The Cr(VI) reduction amounting up to 98.1% could be achieved by replacing Cr_2_O_3_ with (Al_1−x_Cr_x_)_2_O_3_ solid solution. However, in situ stabilized Cr(III) phases as a mixture of (Al_1−x_Cr_x_)_2_O_3_ and Ca(Al_12−x_Cr_x_)O_19_ solid solution hardly reveal any reoxidation. Moreover, the CA_6_ was much more stable than CA and CA_2_, and it did not participate in any chemical reaction with (Al_1−x_Cr_x_)_2_O_3_ solid solution.

## 1. Introduction

Cr_2_O_3_-containing refractories possess remarkable corrosion resistance due to their extremely low solubility and high chemical stability against molten slag. Therefore, they are widely used as lining materials in incinerators, gasifiers, glass furnaces, non-ferrous smelting, etc. [1,2,3,4,5,6]. In addition, refractories as castables have become a popular choice in recent decades because of the energy-saving manufacturing process, convenient for installation and repair works, where binders’ chemistry plays a crucial role [7,8,9]. Calcium alumina cement (CAC) binders are the most widely used since they exhibit fast setting and strength development, stable thermo-mechanical behaviour, and resistance to slag attack [10]. However, Cr_2_O_3_ can oxidize into toxic Cr(VI) products at high temperatures upon reaction with alkali or alkaline earth metals/oxides/compounds under an oxidizing atmosphere [11,12,13]. The Cr(VI) compounds pose a severe threat to humans and the environment since they are toxic, carcinogenic, highly soluble in water, and quickly enter the food cycle [14]. Therefore, it is of significant environmental and practical significance to inhibit the generation of Cr(VI) when applying Al_2_O_3_-CaO-Cr_2_O_3_ castables as lining materials.

The Al_2_O_3_-CaO-Cr_2_O_3_ system was not investigated in detail earlier though numerous Cr(VI) reduction techniques were described for other applications [15,16,17]. Generally, Cr(VI) formation was closely related to the atmosphere and basicity of other components in the Cr_2_O_3_-containing materials [18,19]. For the Cr_2_O_3_-containing refractory linings, since the operating conditions and service atmosphere in a given furnace can hardly be changed in practical production, most related work has focused on Cr(VI) minimization using some additives at high temperatures. The acidic components such as SiO_2_, TiO_2_, Fe_2_O_3_, and P_2_O_5_ can effectively suppress the Cr(III) oxidation during thermal treatment of Cr_2_O_3_-containing refractories [19,20,21,22]. However, these oxide additives usually result in low melting point phases in the matrix, deteriorating either the thermo-mechanical properties or the slag corrosion resistance [23,24]. Previous research indicated that incorporating chromium into solid solution phases can reduce the risk of Cr(VI) formation in the Cr_2_O_3_-containing materials [25,26,27]. For example, the investigation of the Al_2_O_3_-Cr_2_O_3_-CaO-MgO pure system confirmed that composite spinel Mg(Al,Cr)_2_O_4_ could co-exist with CA_2_, where chromium existed in +3 state [25,27]. Again, Wu et al. [28] studied the effect of temperature on Cr(VI) formation in a pure (Al,Cr)_2_O_3_ system with CAC in air atmosphere, where (Al_1−x_Cr_x_)_2_O_3_ was found to be chemically stable against CAC up to 1100 °C, beyond which Ca_4_Al_6_CrO_16_ (hauyne) phase predominantly start from.

Nath et al. investigated the phase evolution of the Al_2_O_3_-CaO-Cr_2_O_3_ refractories castables after treatment at various temperatures, where CaO from cement facilitated the conversion of Cr(III) into Cr(VI) [29]. The main phase of CAC (CA and CA_2_) react with Cr_2_O_3_ in the air to produce CaCrO_4_ and Ca_4_Al_6_CrO_16_ at mid-temperature (700–1100 °C). At the same time, nearly all the Cr_2_O_3_ would convert into (Al_1−x_Cr_x_)_2_O_3_ (0 < x < 1) and Ca(Al,Cr)_12_O_19_ solid solution at 1500 °C, which leads to a significant decrease of Cr(VI) compounds amounts [20,29]. Thus, (Al_1−x_Cr_x_)_2_O_3_ solid solutions having high refractoriness and good chemical stability could be better performing materials with better mechanical properties and slag corrosion resistance [30,31,32,33]. Based on the above research, it can be inferred that substituting Cr_2_O_3_ with (Al_1−x_Cr_x_)_2_O_3_ solid solution as a starting component in the Al_2_O_3_-CaO-Cr_2_O_3_ castables could be a feasible way to inhibit the formation of Cr(VI) at various temperatures, especially at mid-temperature (700–1100 °C), without compromising other properties. However, systematic work relating to the effect of (Al_1−x_Cr_x_)_2_O_3_ solid solution on Cr(VI) formation in Al_2_O_3_-CaO-Cr_2_O_3_ refractory castables is rare.

The present work aims to inhibit the formation of Cr(VI) compounds in Al_2_O_3_-CaO-Cr_2_O_3_ castables by substituting Cr_2_O_3_ with (Al_1−x_Cr_x_)_2_O_3_ solid solution as starting chromium-containing constituent. Firstly, (Al_1−x_Cr_x_)_2_O_3_ solid solutions were pre-synthesized at 1300 to 1650 °C. Secondly, Al_2_O_3_-CaO-Cr_2_O_3_ castables with the pre-synthesized (Al_1−x_Cr_x_)_2_O_3_ solid solution were fabricated and treated at the temperature range of 300–1500 °C in the air since castables would be put to use without firing and a temperature gradient occurs in any furnace linings in actual practice. The phase evolution and Cr(VI) generation of the Al_2_O_3_-CaO-Cr_2_O_3_ castables with temperature and the corresponding mechanism were studied using XRD and related software, SEM, and leaching tests. Furthermore, since the (Al_1−x_Cr_x_)_2_O_3_ and Ca(Al,Cr)_12_O_19_ could be formed in the Al_2_O_3_-CaO-Cr_2_O_3_ castables at high temperature [20], castables with Cr_2_O_3_ were pre-heated at 1500 °C to produce the in situ formed (Al_1−x_Cr_x_)_2_O_3_, whose effect on the Cr(VI) formation for the castables at various temperature was also evaluated.

## 2. Materials and Methods

Tabular alumina (Al_2_O_3_) of various size fractions, 5–3 mm, 3–1 mm, 1–0 mm, and ≤0.045 mm, were procured from Zhejiang Zili Alumina Materials Technology Co., Ltd., Shangyu, China. Reactive α-alumina fines of size fraction ≤ 0.005 mm were purchased from Kaifeng Tenai Co., Ltd., Kaifeng, China. Industrial-grade fused chromium oxide (Cr_2_O_3_) (size ≤ 0.074 mm) was obtained from Luoyang Yuda Refractories Co., Ltd., Luoyang, China. The hydraulic calcium aluminate cement binder, Secar 71 (CA and CA_2_ phases), was procured from Imerys Aluminates, Tianjin, China. An organic defloculant, FS 65 (Wuhan Sanndar Chemical Co., Ltd., Wuhan, China), was used as the dispersant. The detailed chemical composition of raw materials is shown in Table 1.

Cr_2_O_3_ and Al_2_O_3_ fine powders with a mass ratio of 8:17 were dry-mixed, pressed into pellets, and then treated at 1300, 1600, and 1650 °C for 3 h in the air to obtain the mixture of Al_2_O_3_, Cr_2_O_3_ and (Al_1−x_Cr_x_)_2_O_3_ solid solution and pre-synthesized (Al_1−x_Cr_x_)_2_O_3_ solid solution. Thus, obtained pellets were then pulverized to 200-mesh powders before adding them into the castables. The specimen with Cr_2_O_3_ and Al_2_O_3_ powders as initial raw materials was labelled as R, while specimens with (Al_1−x_Cr_x_)_2_O_3_ solid solution pre-synthesized at 1300 °C, 1600 °C, and 1650 °C were designated as S13, S16, and S165, respectively. Specimen R was pre-heated at 1500 °C for 3h (labelled as F15) to produce the in situ formed (Al_1−x_Cr_x_)_2_O_3_, whose effect on the Cr(VI) formation in the castables at various temperatures was also evaluated then. The castables were formulated based on the Andreasen distribution coefficient (q) value of 0.31, and the specific formulation is shown in Table 2. Each batch was dry-mixed for 3 min in a Hobart mixer followed by wet-mixing (4.0 wt% water, 25 °C) for further 3 min, and then castables were moulded in a vibrating table (1 min) into bars of size 160 mm × 40 mm × 40 mm at room temperature. All specimens were cured at 25 °C and 75% ± 5% relative humidity for 24 h in a standard cement maintainer and dried at 110 °C for 24 h in an electric air oven after demoulding. Dried specimens R, S13, S16, S165, and specimen F15 were finally heated in the temperature range of 300–1500 °C for 3h at peak temperature in air.

To figure out relative oxidation, the mechanisms of the Cr(VI) generation in the castables and the corresponding chemical reactions, fine powders of CAC and CA_6_ were mixed with Cr_2_O_3_ and pre-synthesized (Al_1−x_Cr_x_)_2_O_3_ (Table 3). Then, the mixed powders were pressed into Φ20 mm × 20 mm cylindrical specimens under a pressure of 50 MPa. After being treated at 900 °C and 1300 °C for 3 h in the air atmosphere, the phase compositions and microstructures of the specimens were analyzed by XRD and SEM, respectively.

The crystalline phase compositions were identified by X-ray diffraction (XRD) patterns using a X’Pert Pro diffractometer (PANalytical, Almelo, Netherlands) (Copper Kα radiation (λ = 1.5418 Å) at 40 kV/40 mA, step size 0.02 over a 2θ range of 5–90°) and analyzed by the software of X’pert Pro High Score (Philips, Almelo, Netherlands). Lattice parameters were calculated using X’pert Pro High Score (Philips, Almelo, Netherlands) and Celref 2.0 software. Microstructure morphology was analyzed by scanning electron microscopy (SEM, Nova 400 Nano-SEM, FEI Company, Hillsboro, OR, USA) equipped with energy dispersive spectroscopy (EDS, Oxford, High Wycombe, UK).

Cr(VI) leachability was evaluated using the leaching test according to TRGS 613 standard procedure, which is suitable for determining water-soluble Cr(VI) compounds in cement and products containing cement. Leaching specimens were prepared by crushing and grinding thoroughly before passing through a 200-mesh sieve (≤74 μm). Fine samples were stirred with deionized water as a leaching solution using a magnetic stirrer at a speed of 300 rpm for 15 min (at room temperature) with a solid–liquid ratio of 1:20. Then, leachates were obtained through a 0.45-μm membrane filter with a glass fibre by vacuum. The Cr(VI) concentration in the leachates was determined using a colorimetric method. The Cr(VI) can react in acid condition with the 1,5-diphenylcarbazide (DPC) to form 1,5-diphenylcarbazone, a red complex (0.02–0.2 mg/L chrome). Then, the absorbance of the leachates after the DPC method was recorded at 540 nm, using a 722 Vis spectrophotometer (Jinghua Instruments, Shanghai, China).

## 3. Results and Discussion

### 3.1. Pre-Synthesis of (Al_1−x_Cr_x_)_2_O_3_ Powders

**Figure 1:**
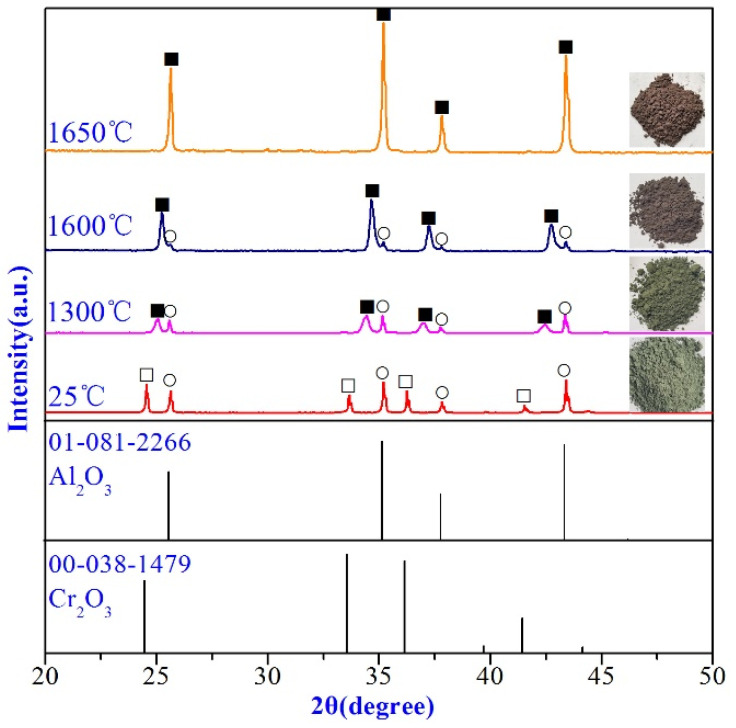
XRD pattern of (Al_1−x_Cr_x_)_2_O_3_ solid solution pre-synthesized at different temperatures. ○-Corundum (Al_2_O_3_), ■-(Al_1−x_,Cr_x_)_2_O_3_ solid solution, □-Eskolaite (Cr_2_O_3_).

The pre-synthesized powders of the (Al_1−x_Cr_x_)_2_O_3_ solid solution at different temperatures are observed by XRD (Figure 1). It could be found that both corundum and eskolaite existed as separate phases after dry mixing at 25 °C. After treated at 1300 °C, the eskolaite disappeared with a noticeable reduction of the peak intensity of corundum, while a new phase identified as (Al_1−x_Cr_x_)_2_O_3_ solid solution was generated. With the increase in the heat treatment temperature, the peak intensity of corundum reduced gradually until disappearance at 1650 °C, while the peak intensity of (Al_1−x_Cr_x_)_2_O_3_ solid solution increased steadily. After treated at temperatures up to 1650 °C, only the (Al_1−x_Cr_x_)_2_O_3_ solid solution could be detected in the specimens. So, it could be inferred that we have added the (Al_1−x_Cr_x_)_2_O_3_ solid solution with the remnant of corundum and eskolaite (sample S13 and S16), while that of S165 is a complete (Al_1−x_Cr_x_)_2_O_3_ solid solution. In addition, the lattice parameters of the (Al_1−x_Cr_x_)_2_O_3_ solid solution were calculated in comparison with Al_2_O_3_ (reference code: JCPDS 01-081-2266, *a* = *b* = 4.7569 Å and *c* = 12.9830 Å) and Cr_2_O_3_ (reference code: JCPDS 00-038-1479, *a* = *b* = 4.9540 Å and c = 13.5842 Å). Since Al_2_O_3_ has smaller lattice parameters than Cr_2_O_3_, the (Al_1−x_Cr_x_)_2_O_3_ solid solution reveals smaller lattice parameters than Cr_2_O_3_. With the increasing temperature, the (Al_1−x_Cr_x_)_2_O_3_ solid solution showed decreasing lattice parameters as more Al_2_O_3_ dissolution is expected at higher temperatures. For example, the lattice parameter *a* = *b* = 4.8607 Å at 1300 °C (for sample S13) decreased to *a* = *b* = 4.8291 Å at 1600 °C (for sample S16).

### 3.2. Cr(VI) Leachability

**Figure 2:**
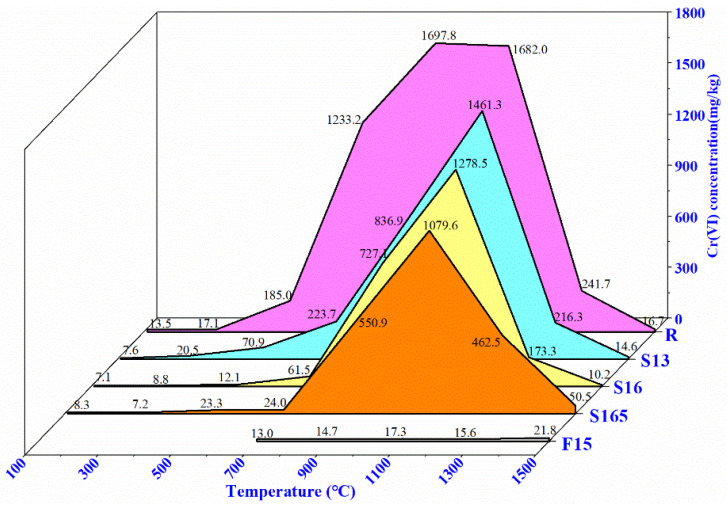
Cr(VI) concentration as a function of temperature in the Al_2_O_3_-CaO-Cr_2_O_3_ castables.

The Cr(VI) concentration in Al_2_O_3_-CaO-Cr_2_O_3_ castables treated at various temperatures was evaluated by leaching test according to the TRGS 613 standard procedure (Figure 2). The details of Cr(VI) reduction compared to the reference specimen R is presented in Table 4. With the addition of the pre-synthesized (Al_1−x_Cr_x_)_2_O_3_ solid solution, a noticeable decrease in the Cr(VI) concentration was observed. The specimens with (Al_1−x_,Cr_x_)_2_O_3_ pre-synthesized at higher temperature exhibited relatively lower Cr(VI) concentrations at the same heat treatment temperature (exception for specimen S165 at 1300 and 1500 °C). For example, at 700 °C, the total amount of Cr(VI) reduced drastically from 1233.2 mg/kg in specimen R (without (Al_1−x_Cr_x_)_2_O_3_) to 223.7 mg/kg in specimen S13 (a reduction of 81.9%), and reduced further to 24.0 mg/kg in specimen S165 (a decrease of 98.1%). However, at 1300 °C, specimen S165 exhibited an even higher Cr(VI) concentration than the reference specimen. Moreover, the temperature corresponding to the maximum Cr(VI) concentration shifted from 900 °C for R to 1100 °C for the pre-synthesized (Al_1−x_Cr_x_)_2_O_3_. The specimen F15, pre-heated at 1500 °C, exhibited extremely low Cr(VI) concentration at all heat treatment temperatures studied. It was concluded that the chromium would present as Cr(III) together within the solid solution of (Al_1−x_Cr_x_)_2_O_3_ and Ca(Al,Cr)_12_O_19_ after the pre-heating treatment at 1500 °C [20]. Therefore, it is plausible that the reoxidation of these stable solid solution phases did not occur. Although the mid-temperature (700–1100 °C) was favourable for Cr(VI) formation, the total amount of Cr(VI) in F15 was still only 13.0-17.3 mg/kg (a decrease of ~98.9–99.1% compared to specimen R). These values are below the allowable Cr(VI) limit of the Environmental Protection Agency (EPA), United States (5 mg/L is equivalent to 100 mg/kg) [34].

### 3.3. Phase Evolution of the Castables

**Figure 3:**
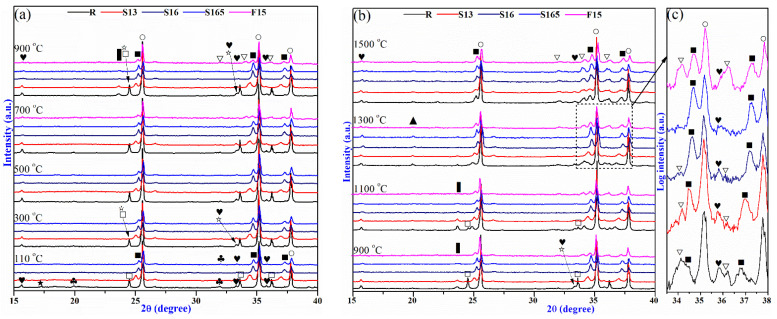
XRD patterns of Al_2_O_3_-CaO-Cr_2_O_3_ castables, (**a**) 110–900 °C, (**b**) 900–1500 ^o^C, (**c**) 1300 °C. ○—Corundum (Al_2_O_3_), ■—(Al_1−x_,Cr_x_)_2_O_3_ solid solution, □—Eskolaite (Cr_2_O_3_), ☆—CaCrO_4_, ▍—Hauyne (Ca_4_Al_6_CrO_16_), ▽—CA_6_ (CaAl_12_O_19_), ♥—NaAl_11_O_17_, ♣—C_3_AH_6_ (Ca_3_Al_2_(OH)_12_).

To study the effect of the pre-synthesized (Al_1−x_Cr_x_)_2_O_3_ solid solution on the phase evolution of the castables, phase compositions of the specimens treated at 110–1500 °C were analyzed (Figure 3). In all samples, the main phase corundum and the NaAl_11_O_17_ impurity could be detected at all temperatures, and hydrate phase C_3_AH_6_ was generated at 110 °C but then disappeared at 300 °C due to dehydration. For specimen R, the CaCrO_4_ phase could be detected at 300 °C, whose peak intensity increased with the increase in temperature from 300 °C to 900 °C but then decreased with further increasing temperature until disappearance at 1300 °C. The Ca_4_Al_6_CrO_16_ was generated at 900 °C, whose peak intensity reached a maximum at 1100 °C but dropped down with further increasing temperature until disappearance at 1500 °C. Moreover, eskolaite existing in the range of 110 °C to 1100 °C reduced in peak intensity with temperature and disappeared at 1300 °C, while the (Al_1−x_Cr_x_)_2_O_3_ solid solution and CaAl_12_O_19_ increased in peak intensity after generating at 1100 °C and 1300 °C, respectively. However, for specimens S13, S16, and S165, no CaCrO_4_ phase was detected at 300–1100 °C, indicating chromium that in the (Al_1−x_Cr_x_)_2_O_3_, the CAC in this temperature range would not oxidize the solid solution. At 900–1300 °C, although the Ca_4_Al_6_CrO_16_ phase was still formed in these specimens with pre-synthesized (Al_1−x_Cr_x_)_2_O_3_, the peak intensity of Cr(VI) compound was much lower compared with sample R. The peak intensity of the Ca_4_Al_6_CrO_16_ phase reached the maximum at 1100 °C in Al_2_O_3_-CaO-Cr_2_O_3_ castables, and therefore, the highest Cr(VI) concentration for the specimens with pre-synthesized (Al_1−x_Cr_x_)_2_O_3_ were detected at 1100 °C (Figure 3b). In general, the substitution of Cr_2_O_3_ with (Al_1−x_Cr_x_)_2_O_3_ in the Al_2_O_3_-CaO-Cr_2_O_3_ castables can almost completely restrict the formation of CaCrO_4_ compounds at 300–1100 °C and effectively lower the Cr(VI) compound Ca_4_Al_6_CrO_16_ formation at 900–1300 °C, which was following the results of Cr(VI) leachability shown in Figure 2. After being treated at 1500 °C, only the corundum (with NaAl_11_O_17_ impurity), the (Al_1−x_Cr_x_)_2_O_3_ solid solution, and the CA_6_ phases were found in specimens R, S13, S16, and S165. The enlarged XRD patterns of the castables (Figure 3c) indicated that samples with (Al_1−x_Cr_x_)_2_O_3_ pre-synthesized at higher temperature exhibited relative lower peak intensity of the CA_6_ phase after being treated at 1300 °C. In addition, specimen F15, which had the same phase compositions as the other four specimens treated at 1500 °C, showed hardly any phase changes with the subsequent heat treatment temperature.

### 3.4. Reaction Mechanism

**Figure 4:**
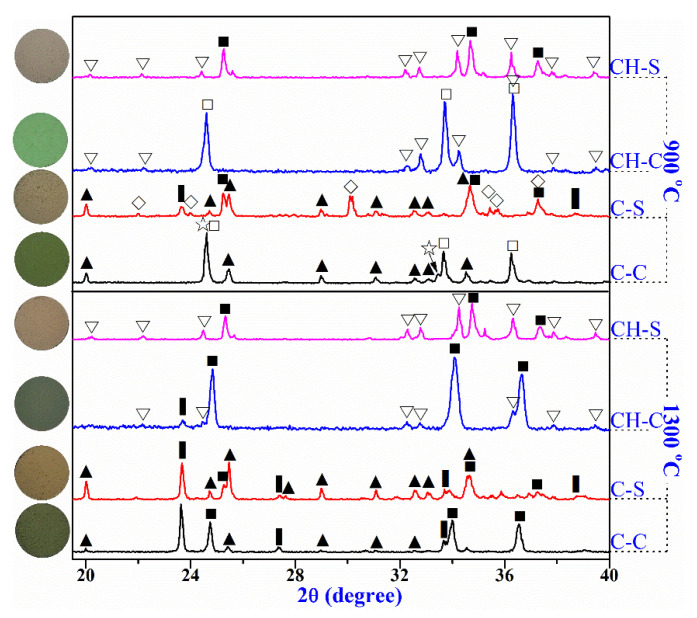
XRD pattern and corresponding images of cylindrical specimens heated at 900 °C and 1300 °C. ■—(Al_1−x_,Cr_x_)_2_O_3_ solid solution. □—Eskolaite (Cr_2_O_3_), ☆—CaCrO_4_, ▍—Hauyne (Ca_4_Al_6_CrO_16_), ▲—CA_2_ (CaAl_4_O_7_), ◇—CA (CaAl_2_O_4_), ▽—CA_6_ (CaAl_12_O_19_).

**Figure 5:**
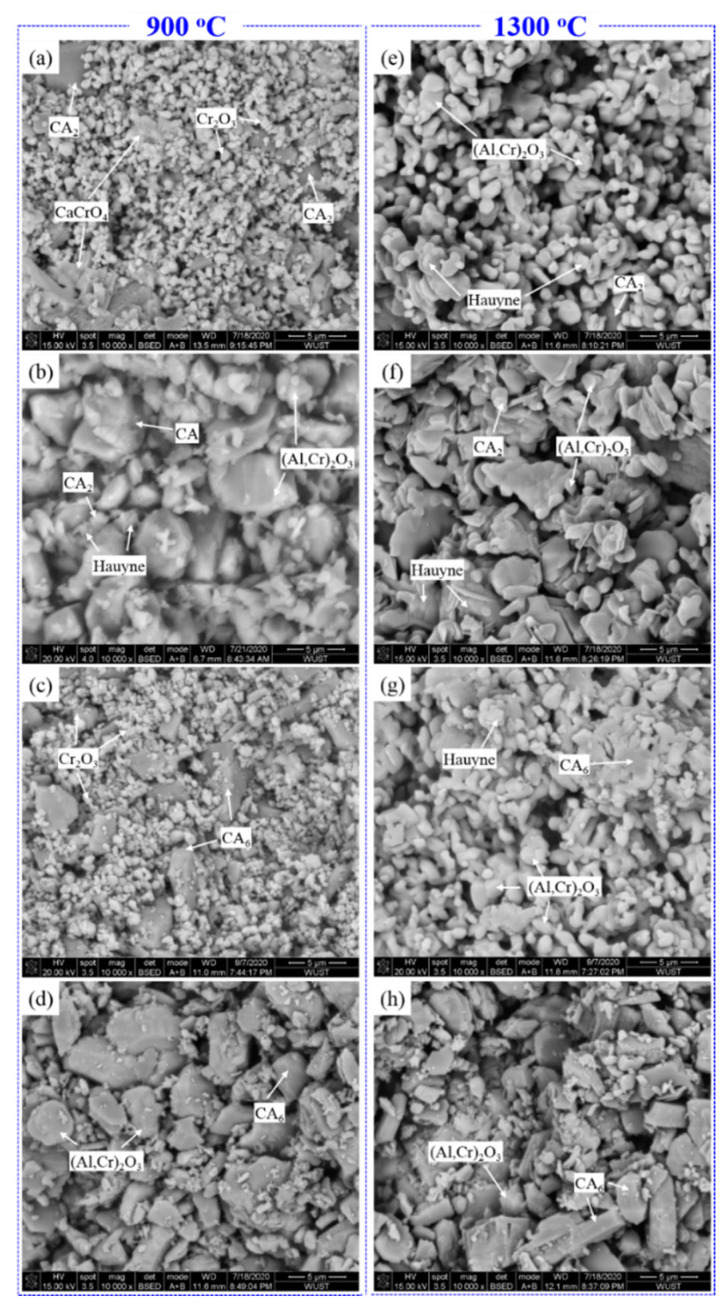
SEM images of cylindrical specimens heated at 900 °C and 1300 °C, (**a**,**e**) C-C; (**b**,**f**) C-S; (**c**,**g**) CH-C; (**d**,**h**) CH-S.

The above results demonstrated that in the Al_2_O_3_-CaO-Cr_2_O_3_ castables, chromium and calcium would exist in the state of Cr_2_O_3_/(Al_1−x_Cr_x_)_2_O_3,_ and CAC/CA_6_, respectively, which affects the formation and concentration of Cr(VI) compounds CaCrO_4_ and Ca_4_Al_6_CrO_16_ at mid-temperature (700–1100 °C). Fine powders of CAC/CA_6_ were mixed with Cr_2_O_3_/(Al_1−x_Cr_x_)_2_O_3_ (pre-synthetized at 1650 °C) to figure out the mechanisms of the Cr(VI) generation in the castables and the corresponding chemical reactions. Then, the mixed powders were treated at 900 and 1300 °C for 3 h in the air; the XRD patterns and SEM microstructure are summarized in Figure 4 and Figure 5, respectively. The plausible chemical reaction equations discussed below in various samples heated at 900 °C and 1300 °C are listed in Table 5. In addition, the qualitative EDS spot analysis (atomic%) was shown in Table 6, corresponding to the fractured surface in Figure 5. Needless to mention that the uneven surface of the specimen would only reveal the non-stoichiometric composition to identify the different phases associated with different morphology.

After being treated at 900 °C, the CA phase disappeared in specimen C-C with the formation of many granular CaCrO_4_ grains (Figure 5a) via reaction 1. However, the sample C-S was still composed of the initial main phases (CA, CA_2_, and (Al_1−x_Cr_x_)_2_O_3_) (Figure 5b) in addition to forming minute amounts of Ca_4_Al_6_CrO_16_ (reaction 2). As the heat treatment temperature increased to 1300 °C, plenty of chrome-hauyne and (Al_1−x_Cr_x_)_2_O_3_ solid solution (Figure 5e) were generated in specimen C-C (via reactions 3–5), accompanied by the disappearance of CA and significant reduction in CA_2_ phase, while specimen C-S possessed relative lower peak intensity of Ca_4_Al_6_CrO_16_ although it had similar phases as C-C. Combining the observations of Cr(VI) in Figure 2, with the phase evolution results (Figure 3 and Figure 4), it can be deduced that compared with Cr_2_O_3_, the (Al_1−x_Cr_x_)_2_O_3_ solid solution was more stable that would not form CaCrO_4_ and could effectively hinder the Ca_4_Al_6_CrO_16_ formation when contacted with CAC. Therefore, the substitution of Cr_2_O_3_ with (Al_1−x_Cr_x_)_2_O_3_ can effectively lower the Cr(VI) concentration of the castables after being treated at various temperatures (Figure 2). Furthermore, the castables with (Al_1−x_Cr_x_)_2_O_3_ pre-synthesized at higher temperature exhibited lower Cr(VI) concentration, implying that the stability of the (Al_1−x_Cr_x_)_2_O_3_ improved gradually with the Al_2_O_3_ proportion in the solid solution. In addition, in comparison with the CA_2_ phase, CA was more likely to react with Cr_2_O_3_/(Al_1−x_Cr_x_)_2_O_3_ resulting in the formation of Cr(VI) compounds.

For specimens CH-C, no new phases occurred after heat treatment at 900 °C, and only a minuscule amount of chrome-hauyne was generated at 1300 °C (Figure 5g) via Eqs. 6, which also produced Al_2_O_3_ that subsequently interacted with Cr_2_O_3_ to develop the (Al_1−x_Cr_x_)_2_O_3_ solid solution via Eqs. 5. It is worth mentioning that no changes in the phase compositions were detected in specimen CH-S after heat treatment at both 900 °C and 1300 °C (Figure 4). These observations demonstrated that calcium in CA_6_ was much more stable than in CA and CA_2_, which only caused slight oxidation of Cr_2_O_3_ and would not take chemical reaction with (Al_1−x_Cr_x_)_2_O_3_ solid solution. Therefore, specimen F15, in which chromium and calcium existed in (Al_1−x_Cr_x_)_2_O_3_ and CA_6_, respectively, showed no changes in phase composition and extremely low Cr(VI) concentration at various heat treatment temperatures. In the Al_2_O_3_-CaO-Cr_2_O_3_ castables, CA_6_ could be generated from the reaction between CAC and Al_2_O_3_ powders in the matrix at 1300 °C (Figure 4). However, for specimen S165, since no Al_2_O_3_ existed in the (Al_1−x_Cr_x_)_2_O_3_ powder pre-synthesized at 1650 °C, the calcium would still exist as CA and CA_2_ rather than CA_6_ at 1300 °C. As a result, specimen S165 possessed an even higher Cr(VI) concentration than the reference specimen R at 1300 °C, suggesting that CA and CA_2_ can more easily react with (Al_1−x_Cr_x_)_2_O_3_ to produce Ca_4_Al_6_CrO_16_ compared with CA_6_.

## 4. Conclusions

In the present work, (Al_1−x_Cr_x_)_2_O_3_ solid solution was pre-synthesized at a different temperatures for the inhibition of the formation of Cr(VI) in Al_2_O_3_-CaO-Cr_2_O_3_ castables was systematically investigated. The summarized conclusions are as follows:

(1) Compared with Cr_2_O_3_, the stability of the (Al_1−x_Cr_x_)_2_O_3_ solid solution in contact with CAC was much higher. Furthermore, the substitution of Cr_2_O_3_ with (Al_1−x_Cr_x_)_2_O_3_ in the Al_2_O_3_-CaO-Cr_2_O_3_ castables can completely inhibit the mid-temperature (300–1100 °C) formation of Cr(VI) compound CaCrO_4_ and relatively higher temperature Cr(VI) phase Ca_4_Al_6_CrO_16_ (hauyne) drastically reduced at 900 to 1300 °C. Therefore, replacing Cr_2_O_3_ with (Al_1−x_Cr_x_)_2_O_3_ can effectively lower the Cr(VI) concentration of the castables after being treated at various temperatures, and a reduction in Cr(VI) amounts up to 98.1% with (Al_1−x_Cr_x_)_2_O_3_ addition could be achieved. Most importantly, Cr(III) present within the in situ (Al_1−x_Cr_x_)_2_O_3_ and Ca(Al,Cr)_12_O_19_ solid solution phases showed maximum reoxidation resistance and thus need further investigation.
(2) In comparison with the CA_2_ phase, CA was more likely to react with Cr_2_O_3_/(Al_1−x_Cr_x_)_2_O_3_, resulting in Cr(VI) compound formation. Simultaneously, calcium in CA_6_ was much more stable than in CA and CA_2_, which only caused slight oxidation of Cr_2_O_3_ and would not undergo a chemical reaction with (Al_1−x_Cr_x_)_2_O_3_ solid solution. Thus, incorporating some Al_2_O_3_ powders in the matrix of the Al_2_O_3_-CaO-Cr_2_O_3_ castables to form CA_6_ at a temperature above 1300 °C was also essential for inhibiting Cr(VI) formation when using (Al_1−x_Cr_x_)_2_O_3_ solid solution as a substitute for Cr_2_O_3_.

## Figures and Tables

**Table 1:** The chemical composition of raw materials (wt%).

Raw Materials	SiO_2_	Al_2_O_3_	CaO	Fe_2_O_3_	MgO	Na_2_O	K_2_O	Cr_2_O_3_
Tabular alumina	0.08	99.30	-	0.02	-	0.28	-	-
Reactive α-alumina	0.28	98.87	0.07	0.13	0.12	0.10	0.005	-
Fused chromium oxide	0.82	0.59	0.38	0.73	0.27	0.14	0.01	94.02
Calcium aluminate cement	0.40	70.6	28.4	0.20	-	-	-	-

**Table 2:** The formulation of fine powders undergoing reaction within castables (30wt% of total).

Code	Aggregates Al_2_O_3_ (wt%)	Fine Powders (wt%)				Pre-Treatment Temperature (°C)
		Al_2_O_3_	Cr_2_O_3_	CAC	(Al_1−x_,Cr_x_)_2_O_3_	
R	70	17	8	5	-	-
F15	70	17	8	5	-	In situ treated at 1500
S13	70	-	-	5	25	(Al_1−x_,Cr_x_)_2_O_3_ made at 1300
S16	70	-	-	5	25	(Al_1−x_,Cr_x_)_2_O_3_ made at 1600
S165	70	-	-	5	25	(Al_1−x_,Cr_x_)_2_O_3_ made at 1650

Note: 0.1 wt% additional organic dispersant was added to each formulation to make the castables. CAC designates calcium aluminate cement (Here, a commercial Secar 71 cement was used). Each batch contains 8 wt% of Cr_2_O_3_.

**Table 3:** The formulation of cylindrical specimens (wt%).

Specimens	CAC	CA_6_	Cr_2_O_3_	(Al_1−x_,Cr_x_)_2_O_3_
C-C	50		50	
C-S	50			50
CH-C		50	50	
CH-S		50		50

Note: CAC (Secar 71) contains mixture of CaAl_2_O_4_ (63%) and Ca_2_Al_4_O_7_ (35%), CA_6_ = CaAl_12_O_19_.

**Table 4:** Relative Cr(VI) reduction (%) of specimens compared to R at different temperatures.

Specimens	Temperature (°C)							
	110	300	500	700	900	1100	1300	1500
S13	43.7	−19.5	61.7	81.9	16.1	21.2	10.5	12.6
S16	47.4	48.6	93.4	95.0	57.2	24.0	28.0	38.7
S165	38.5	58.0	87.4	98.1	67.6	35.8	−91.4	−202.4
F15	-	-	-	98.9	99.1	99.0	93.5	−30.8

**Table 5:** Chemical reaction equations in cylindrical specimens.

Specimens	900 °C	1300 °C
C-C	(1)	(3) (4) (5)
C-S	(2)	(2)
CH-C	-	(5) (6)
CH-S	-	-
${4\mathrm{CaAl}}_{2}O_{4}{\;+\;2\mathrm{Cr}}_{2}O_{3}{\;+\;3O}_{2}\to {4\mathrm{CaCrO}}_{4}{\;+\;4\mathrm{Al}}_{2}O_{3}$		(1)
${16\mathrm{CaAl}}_{2}O_{4}{\;+\;y({\mathrm{Al}}_{1-x},\mathrm{Crx})}_{2}O_{3}{\;+\;3O}_{2}\to {4\mathrm{Ca}}_{4}{\mathrm{Al}}_{6}{\mathrm{CrO}}_{16}{\;+\;(y\;+\;2)\mathrm{Al}}_{2}O_{3}$		(2)
${16\mathrm{CaAl}}_{2}O_{4}{\;+\;2\mathrm{Cr}}_{2}O_{3}{\;+\;3O}_{2}\to {4\mathrm{Ca}}_{4}{\mathrm{Al}}_{6}{\mathrm{CrO}}_{16}{\;+\;4\mathrm{Al}}_{2}O_{3}$		(3)
${16\mathrm{CaAl}}_{4}O_{7}{\;+\;2\mathrm{Cr}}_{2}O_{3}{\;+\;3O}_{2}\to {4\mathrm{Ca}}_{4}{\mathrm{Al}}_{6}{\mathrm{CrO}}_{16}{\;+\;20\mathrm{Al}}_{2}O_{3}$		(4)
${(1\;-\;x)\mathrm{Al}}_{2}O_{3}{\;+\;\mathrm{xCr}}_{2}O_{3}\to {\left({\mathrm{Al}}_{1-x},\mathrm{Crx}\right)}_{2}O_{3}$		(5)
${16\mathrm{CaAl}}_{12}O_{19}{\;+\;2\mathrm{Cr}}_{2}O_{3}{\;+\;3O}_{2}\to {4\mathrm{Ca}}_{4}{\mathrm{Al}}_{6}{\mathrm{CrO}}_{16}{\;+\;84\mathrm{Al}}_{2}O_{3}$		(6)

**Table 6:** Examples of qualitative EDS spot analysis of the samples (atomic%) for identifying various phases in Figure 5.

Phase	Al	Ca	Cr	O
CaCrO_4_	-	28.36	41.27	30.37
Ca_6_Al_4_CrO_16_	34.27	18.58	6.34	40.82
CaAl_2_O_4_	40.39	16.94	-	42.68
CaAl_4_O_7_	49.87	4.13	-	46.00
(Al,Cr)_2_O_3_	48.68	-	15.27	36.05

## Data Availability

Data sharing not applicable.
